# A Qualitative Investigation of the Experiences of Tobacco Use among U.S. Adults with Food Insecurity

**DOI:** 10.3390/ijerph19127424

**Published:** 2022-06-16

**Authors:** Jin E. Kim-Mozeleski, Susan J. Shaw, Irene H. Yen, Janice Y. Tsoh

**Affiliations:** 1 Prevention Research Center for Healthy Neighborhoods, Department of Population and Quantitative Health Sciences, Case Western Reserve University, Cleveland, OH 44106, USA; 2 Center for Community Health Equity Research, Department of Health Promotion and Policy, School of Public Health and Health Sciences, University of Massachusetts, Amherst, MA 01003, USA; sjshaw@umass.edu; 3 Public Health, University of California, Merced, CA 95343, USA; iyen@ucmerced.edu; 4 Department of Psychiatry and Behavioral Sciences, University of California, San Francisco, CA 94143, USA; janice.tsoh@ucsf.edu

**Keywords:** tobacco use, food insecurity, poverty, disparities, qualitative

## Abstract

Background: Low-income U.S. adults experiencing food insecurity have a disproportionately high prevalence of cigarette smoking, and quantitative studies suggest that food insecurity is a barrier to quitting. To guide effective tobacco control strategies, this study aimed to understand the experiences, perceptions, and context of tobacco use and cessation among low-income populations experiencing food insecurity. Methods: We conducted in-depth, semi-structured interviews with 23 adults who were currently smoking cigarettes and were experiencing food insecurity, mostly living in rural settings. Participants were recruited through food-pantry-based needs assessment surveys and study flyers in community-based organizations. The interview guide explored participants’ histories of smoking, the role and function of tobacco in their lives, their interest in and barriers to quitting, as well as lived experiences of food insecurity. We used reflexive thematic analysis to analyze transcribed interviews. Results: Within a broader context of structural challenges related to poverty and financial strain that shaped current smoking behavior and experiences with food insecurity, we identified the following five themes: smoking to ignore hunger or eat less; staying addicted to smoking in the midst of instability; smoking being prioritized in the midst of financial strain; life stressors and the difficulty of quitting smoking and staying quit; and childhood adversity at the intersection of food insecurity and tobacco use. Conclusion: The context of tobacco use among adults with food insecurity was highly complex. To effectively address tobacco-related disparities among those who are socially and economically disadvantaged, tobacco control efforts should consider relevant lived experiences and structural constraints intersecting smoking and food insecurity. Findings are applied to a conceptualization of clustering of conditions contributing to nicotine dependence, food insecurity, and stress.

## 1. Introduction

Cigarette smoking in the U.S. has become disproportionately concentrated among individuals with socioeconomic disadvantage, leading to tobacco-related health disparities [1]. A socioeconomic gradient in combustible tobacco use—of which cigarette smoking is the most common form—is now more evident than in past decades with respect to household income, education attainment, and type of health insurance coverage [2,3]. Socioeconomically disadvantaged persons are more likely to smoke cigarettes and are less likely to quit successfully than the general population [4,5], and this is in spite of broader access to evidence-based cessation treatments.

Populations with socioeconomic disadvantage face a greater burden of financial strain and unmet needs. Financial strain in particular has been linked with higher prevalence of cigarette smoking [6,7]. Food insecurity, which occurs when access to enough food for an active and healthy life is limited by a lack of money or other resources [8], is a social determinant of smoking status [9]. Up to half of U.S. adults with food insecurity smoke cigarettes, and tobacco prevalence increases as food insecurity grows more severe [10]. A significant body of research, including nationally representative studies, clinical studies, and community-based studies, has shown that food insecurity is uniquely related to current cigarette smoking [11]. However, lived experiences of individuals at the intersection of smoking and food insecurity are not well understood. Especially for low-income, rural communities, qualitative approaches are needed to provide deeper insights that can assist researchers and interventionists to identify contextual drivers of smoking behavior and barriers to cessation in this population.

More and more tobacco cessation interventions, in both health care and community settings, aim to integrate and address social needs [12,13]. Despite these important advances, tailored approaches are needed to meet the unique circumstances of marginalized groups who are disproportionately impacted by tobacco-related health disparities [14,15]. For instance, the extant literature has described experiences of smoking-induced deprivation, in which tobacco expenditures compete with spending on food and other necessities [16]. Yet, these relationships are multifaceted and not unidimensional. Mills and colleagues recently drew on systems dynamics science to elucidate the complex factors that maintain socioeconomic disparities in smoking. The authors described reinforcing feedback loops amongst financial strain, stress and anxiety, lack of control, and smoking in addition to policy-level drivers [17]. 

The current study aimed to qualitatively examine the perceptions, experiences, and context of smoking and smoking cessation among adults with socioeconomic disadvantage who experience food insecurity. This investigation fills an important gap by illuminating how low-income people who smoke cigarettes view their smoking and by contextualizing their smoking and cessation behavior in light of food insecurity and related socioeconomic stressors. 

## 2. Methods

This study took place in largely rural communities in western Massachusetts. We used purposive sampling to recruit adults with recent food insecurity experiences who currently smoke or formerly smoked cigarettes. Participants were predominantly recruited from needs-assessment surveys, flyers, and word-of-mouth in local food pantries although recruitment efforts included flyers at other community-based services organizations, advertisements on local bus routes, and online advertisements through Craigslist (a classified advertisement website). Participants who provided informed consent were interviewed by telephone between June and August 2019. Semi-structured interviews lasted one hour on average (range of 27 to 150 min), and participants were provided with a USD 50 gift card to a grocer of their choice as an acknowledgment for their participation. 

The semi-structured interview guide featured questions about their current life circumstances, current and past cigarette smoking behaviors (and other tobacco use, if relevant), current and past interests in cessation, tobacco budgets and expenditures, and current and past experiences of food insecurity. We provide examples of the most relevant interview questions and probes in Table 1. Audio-recorded interviews were professionally transcribed verbatim, and each transcript was checked for accuracy. In alignment with the research questions of the current study, we analyzed data from a subset of the full study sample, of 23 participants who were currently smoking. Participant characteristics are described in Table 2. 

The first author and two research assistants read the transcripts to initially identify and discuss potentially meaningful segments across all of the transcripts. The first author used Braun and Clarke’s reflexive thematic analysis [18,19] to code and analyze themes across the interviews. Reflexive thematic analysis is a theoretically flexible approach that relies on the researcher’s active role in the production of meaning from the data and is well-suited for qualitative research aimed at understanding people’s experiences and perceptions. Each step of six-step process of thematic analysis was followed, with codes identified through an inductive and deductive process, which then informed the initial set of themes [1,2]. Numerous discussions amongst all authors informed the revised themes presented in this study. All study procedures were approved by the university Institutional Review Board. 

In the following Results section, we use the terms “smoking” or “tobacco use” to refer to the use of cigarettes or other commercially sold combustible tobacco products. 

## 3. Results

### Structural Marginalization, Food Insecurity, and Tobacco Use

The experience of poverty and structural marginalization serves as the overarching context of the five themes that emerged from in-depth interviews. Participants’ experiences of food insecurity and tobacco use were shaped by intersecting structural factors that included housing insecurity, low access to reliable transportation, living on disability benefits (i.e., poor health status and fixed incomes), and navigating food assistance and other areas of bureaucracy to make ends meet. Participants’ housing conditions were varied and at times extreme, such as living in a foreclosed home, living in a trailer without running water, and living out of a car without a working heater even during the winter months. 

Each of the five thematic areas below is shaped by this overarching context of structural marginalization. We recognize at the outset that the perceptions, experiences, and context of tobacco use and cessation in this sample of adults with food insecurity are difficult to separate into discrete themes. The overlap and interactions among themes are a noteworthy feature of the findings, as they illustrate that smoking and food insecurity are both inextricably connected to structural disadvantage.

**Theme 1: Smoking to ignore hunger or eat less**

The relationship between smoking and eating was multifaceted. Smoking was relied upon as a strategy to feel less hungry in the moment, whether it was out of necessity to stretch small amounts of food or smoking to generally eat less as a way to manage body weight. One participant, “Julie” (all names are pseudonyms), was living with diabetes. She was unemployed and looking for a job while doing an unpaid internship to improve her future job prospects. She shared custody of her daughter with her ex-partner and was living in subsidized housing through Section 8 (a housing/rental assistance program for low-income households). Julie described how she relied on smoking to suppress hunger:

*[Smoking] takes away my appetite. Because of growing up and not having food and stuff like that, I kind of don’t necessarily feel hunger, like I block it out. But it’s obviously there. My body’s hungry, it would like to eat. But on the times that I do feel hungry, if I smoke a cigarette, it goes away…I think I smoke more on the days I don’t eat, specifically if they’ve been multiple days, like more than one day in a row that I haven’t eaten. I definitely smoke more to combat that.*

(Julie, female participant in mid 40s)

While smoking may allow Julie to ignore feelings of hunger, it is not a true substitute for the food that she needs to subsist. Smoking helped her eat less when she is hungry, but it counteracted her diabetes medication, which causes low blood sugar without adequate caloric intake. 

Another participant, “Lisa”, dealt with irritable bowel syndrome, which affected her options at the food pantry. For Lisa, smoking cigarettes temporarily staved off hunger and bought her time to figure out what she had available to eat:

*So the instant gratification of the cigarette makes it so that I automatically stop feeling hungry at that moment. And it’ll last for, I think about an hour. I’ll usually get an hour, like if I don’t have food [by] then, I’ll smoke again. If I have something to eat [in the house], I might eat.*

(Lisa, female participant in mid 30s)

Both Julie and Lisa regularly experienced very low food security (or severe food insecurity), for which hunger and skipping meals due to lack of money are hallmarks. 

For other participants, smoking was a way to suppress hunger for weight management. “Jasmine” was living with diabetes and hypertension and had concerns about her weight. Jasmine tried to quit smoking following her doctor’s advice, which led to weight gain, while managing daily stressors.

*I’m already a chubby girl, so it was just like I was really huge within two months. It was insane… And it became a greater risk factor, you know, having the diabetes and the hypertension and then the extra added weight. So it was just like ‘Well, you want me to lose weight, it’s kind of hard to do that if I’m not smoking.’ And then I tried the gym, and that just wasn’t working for me. It didn’t fit into my time. My life time of just trying to work, the kids, appointments, meetings… And so I started smoking again. I started smoking because I was stressed out, and I started smoking, and I started losing the weight all over again. It’s crazy because the cigarettes—the cigarettes sometimes replace a meal for me, almost.*

(Jasmine, female participant in mid 30s)

Jasmine perceived weight gain, which was noticeable and rapid, to be more detrimental to her health than smoking. The relatively quick weight loss after restarting smoking thus reinforced a dependence on smoking to maintain weight in the midst of diet-sensitive chronic conditions, which already tend to be common among people experiencing food insecurity.

**Theme 2: Staying addicted to smoking in the midst of instability**

Many participants described symptoms of addiction to cigarettes: they continued to smoke despite known negative consequences for self and others (from health to relationships); they described a sense of helplessness and loss of control over their smoking; and, as discussed further in the following theme, they were willing to expend a significant amount of money and effort to obtain cigarettes. Yet, in keeping with an American commitment to individual responsibility for one’s health behaviors, participants also struggled with guilt about their smoking. Participants wove together multilayered experiences of guilt around the cost of cigarettes, the health risks posed by smoking for themselves and their families, and their failure to quit smoking and stay quit. For many, guilt formed another stressor that combined with other stressors described above to exacerbate the urge to smoke. 

In its consistent compulsion, smoking seemed to provide a stable component in participants’ lives, which were in many ways precarious and uncertain. For example, “Mel” had caretaking responsibilities of her aging parents whom she lived with, and they were in poor health. Since her income as a freelance writer fluctuated, she did not participate in the Supplemental Nutrition Assistance Program (SNAP) anymore because her eligibility varied, and the documentation process became difficult to navigate. For a time, she relied on “dumpster diving” outside of restaurants as a way to procure food not just for herself but for her household but stopped when she became worried about the potential health consequences to her aging parents of eating discarded food. She depended on cigarettes in order to do her work as a writer, which required sitting and concentrating for long periods of time:

*[I feel] a little bit guilty because I know that I shouldn’t be smoking cigarettes. I know that. And like, I also have physical problems that are worse because I smoke. And like I know that, like, I’m making that choice even though, yeah, I’m addicted to nicotine but I’m making a choice not to give it up so I feel guilty.*

(Mel, female participant in early 30s)

By perceiving her addiction to nicotine as being a choice, Mel compounded the guilt she felt around smoking. Julie, the participant with diabetes, sometimes subsisted on peanut butter from the food pantry, and reflected that while she regularly went without food herself to ensure that her daughter would have enough to eat, she made tradeoffs for herself to continue to smoke:

*I think an important point that I would like to make is that I sacrifice food [for myself] for my daughter, but for some reason I would not sacrifice cigarettes for food for myself, and I don’t know why that is.*

Indeed, Julie continued to smoke despite her doctors’ warnings of its negative health effects, including a potential health scare. She reprimanded herself for smoking but continued to smoke:

*I always say, ‘Yeah, someday [I’ll quit], but today’s not that day.’ Back in the end of February, I believe, I was sick and I went to—I think it was one of the urgent care places. And they did x-rays and found spots on my lungs. And every time I lit up a cigarette, I’m thinking to myself, yeah, what are you doing? This isn’t okay. But I would still light up and smoke the cigarette.*

(Julie, female participant in mid 40s)

Participants like Julie who have children struggled to reconcile their addiction to smoking with their responsibility to serve as a role model for their children. For example, “Anne” was a single mother who had a history of child custody battles with her ex-partner and was living in Section 8 housing with her son at the time of her interview. She recently lost her SNAP benefits because she forgot to complete her recertification paperwork in the midst of her increasingly busy schedule working as a personal care attendant, picking up extra hours at a fast food restaurant, and deciding to go back to school to finish her associate’s degree. In response to an interviewer’s question of whether others ever comment on her smoking, Anne mentioned her young son:

*It’s basically so when my son mentions anything, like he’s told me once that he wants me to quit smoking cigarettes. Just once. But the thing that twinges at my heart strings or makes me feel really crappy is when I’m going to go outside to have a cigarette and he’s like ‘Mommy, are you going out to have a cigarette?’ He’s 7 years old. He shouldn’t—cigarette is not an easy word to say. It shouldn’t be a normal word in his vocabulary and that kind of makes me feel like a really shitty mom. I know that’s not the case, but you know.*

(Anne, female participant in late 20s)

After fighting for custody, Anne worked numerous jobs to provide for her son. Forgetting to complete a task on time had considerable consequences with the lapse in SNAP benefits. She felt guilty that she continued to smoke and tried to protect her son’s health by always smoking outside, but her son’s observations of even this behavior tugged at her heart. 

“Jan” is another single mother who has two young daughters. She was receiving an amount of child support that disqualified her from SNAP benefits, but child support was her only source of income while she was between jobs. Jan reported:

*Like I probably will take [USD] $20 out of [child support] a week. I’ll buy a couple of packs out of the money but I try—I don’t like doing that because then I feel even guiltier about the guilt I feel on top of when I go smoke a cigarette and the kids are ‘No. You’re my mom. I don’t want you to smoke.’ And also using some of the money that’s designated for their care.*

(Jan, female participant in mid 30s)

Jan’s addiction to smoking was apparent in her willingness to use her child support money to pay for cigarettes and in the layers of guilt she felt about doing so on top of the guilt caused by smoking itself. Participants smoked to manage the stress of their economic instability and their responsibilities as caregivers of their children or aging parents. Guilt around their addiction exacerbated these stresses and was compounded by an understanding of health behavior as a personal responsibility or individual choice.

**Theme 3: Smoking gets prioritized in the midst of financial strain**

This theme of prioritizing smoking in the midst of financial strain shares parallels with the previous theme delineating addiction to smoking in the face of instability. Participants were highly aware of tobacco costs and dedicated substantial time and effort towards obtaining cigarettes or making them more affordable. Participants adopted several cost-saving strategies, such as switching to cheaper brands or switching to rolling their own tobacco and, in one instance, spending an hour each day to roll them. Cigarette expenses were considered a fixed item in participants’ budgets and had already been reduced as much as possible. 

Participants were exacting in managing their tobacco costs, as exemplified by “John”, who was in his early 50s, single without any children, and was looking for a job while sharing an apartment with friends.

Interviewer: *I know you said you’re unemployed right now and you’re on a limited income, but how do cigarettes factor into your budget? How do you organize your budget?*

John: *Well, I actually roll my own cigarettes now, so it’s cheaper. It’s a lot cheaper actually. If I had to pay for regular cigarettes, I couldn’t do it… I spend about $100 a month… I was spending almost $100 a week. Because I roll them it’s only like $100 for the whole month… I’m pretty much locked into the same price all the time, you know?*

John was a pack-a-day cigarette smoker for many years and switched to rolling his own cigarettes as a way to cut costs while maintaining his consumption level. Though he received SNAP benefits, they regularly ran out towards the end of the month, and he struggled to eat more than one meal per day. Thanks to previous experiences working as a chef, John was skilled in making appetizing meals for himself with few ingredients. He explained, *“I decided [to] basically eat one meal a day,”* illustrating the tradeoff between the financial cost of smoking and food, which was necessary because smoking helped with stress.

*People that are in my situation have financial burdens with the food and everything. But smoking is a big thing and at one point, yes, it’s expensive and it takes a lot out of your budget. But it also takes the worry away too.*

(John, male participant in early 50s)

Anne, the personal care attendant, worked several jobs to cover her own and her son’s expenses and so that she would always have money for cigarettes. She said, *“I’ve never been good at saving money, and so that’s like my hardest struggle is trying to save money so that if my car breaks down I have the money to fix it.”* This exemplifies that one event out of the ordinary can be detrimental to financial situations that have little room for error. In light of this difficulty, Anne was somewhat embarrassed about budgeting for her cigarettes:

Anne: *So I think cigarettes are the only thing that I, for sure, make sure to budget every month. Like that is the constant.*

Interviewer: *You make a budget?*

Anne: *Yes. That’s like the only thing that I can successfully budget. And it’s sad to say because I should be able to successfully budget anything if I can budget that into my very little income that I was having.*

Buying cigarettes by the carton allowed participants to treat their cigarette purchases like other monthly expenses. For instance, “Tina” was living with her son in a foreclosed house in her ex-partner’s name. She received Supplemental Security Income (SSI) after suffering a stroke, but prior to that, her monthly routine included trips out of state to buy cigarettes:

*Well, I just counted it like a bill. So on the first, you know, when I got paid, I would go to New Hampshire and buy them for the month, so basically I just acted like it was the cable bill, but it was my cigarette habit bill.*

(Tina, female participant in mid 40s)

Some participants such as Tina would drive to a neighboring state where tobacco taxes were lower in order to save money on cigarettes. It was not uncommon for participants to drive up to an hour for cheaper cigarettes.

Not having enough money for cigarettes also caused stress, while cutting down on smoking increased cravings to smoke. Mel, the freelance writer, explained how her unpredictable income affected her smoking behavior:

*I do try to smoke less when I know I’m going to be like completely broke…Like, I try to. It’s hard. Like I often get stressed out about the fact that I don’t have enough money for everything I need. And that just makes me want to smoke more.*

(Mel, female participant in early 30s)

Mel tried to moderate her smoking behavior at times when she anticipated having less money and acknowledged that those lean times are also a trigger to increase cigarette consumption as a means to manage stress. This nuances the notion of smoking-induced deprivation, which tends to be viewed unidirectionally and may therefore be incomplete in its conceptualization. For instance, “Edna”, who was in her late 60s, lived alone in her own home and had an advanced professional degree but had been unable to work after an illness many years ago. She had a car in her driveway that she had not been able to drive because the registration was lapsed and now lived on a fixed income with disability (SSI). She sometimes sold household items to be able to buy cigarettes and stated: *“If I could buy cigarettes all the time, it probably wouldn’t be such a priority.”* This illustrates that the scarcity of cigarettes due to one’s financial situation impacted the relative importance of having cigarettes. 

In this theme, smoking was prioritized in the midst of financial strain, and participants engaged a variety of strategies to reduce the cost of smoking and made tradeoffs in order to continue to smoke. These trade-offs came at a considerable cost, however, as participants spent gas money and time to travel to buy cheaper cigarettes elsewhere. Other participants felt shame for using resources that ought to have supported their children. Furthermore, the stress from financial concerns, including worrying about food and subsistence needs, led to smoking for stress relief or a break from worrying about finances.

**Theme 4: Life stressors and the difficulty of quitting smoking and staying quit**

Most participants were currently interested in quitting, and many described prior experiences with evidence-based cessation methods, including the use of varenicline, bupropion, and nicotine-replacement therapy. They recalled prior periods of abstinence, followed by various triggering and stressful circumstances that led to relapsing to smoking. For example, “Lisa”, who lived with irritable bowel syndrome, had quit smoking in preparation for a back surgery. She maintained abstinence for six months before relapsing:

*After I quit the last time before the surgery that should have been it. But then we moved and all of the money [I saved] was stolen and it could have been stolen by someone we lived with. So that was stressful because these are people I was supposed to trust. And once we moved into our own apartment, the stress of moving into our own apartment died down I quit again. And it was for a few months. And we found out our friend needed a place to stay and he was going to be staying with us and I immediately started smoking again because I have social anxiety. So a really small apartment with an extra person just kind of put me in a place where it wasn’t my normal self. So smoking helps.*

(Lisa, female participant in mid 30s)

Lisa expected to be able to maintain abstinence after the back surgery, but housing insecurity and a theft led to relapse. Many participants described prior unsuccessful quit attempts whose failures they attributed to social and environmental factors. Some, therefore, wished for a more drastic change from their present circumstances so that they could focus on quitting. Anne, the personal care attendant who was returning to school for an associate’s degree, wanted a break from her day-to-day responsibilities in order to have a reasonable chance to quit:

*Before this last time that I attempted to quit, I knew that I would be able to quit if I could have three days off, of no work, no school, no responsibility, and just sleep because the first three days are the hardest. So I had asked my sister like, ‘Hey, I want to try and quit smoking. Is there any way that I can have you have [my son] for a day and a half so I can just sleep? And she’s like ‘No, that’s not good for you or for him.’ And like she doesn’t understand.*

(Anne, female participant in late 20s)

Anne attempted to make the plans she needed in order to get through the most acute phases of nicotine withdrawal, but her family/social support was inadequate to meet this need. Notably, Anne’s plans for quitting did not include any pharmaceutical support to manage symptoms of withdrawal. 

Another participant wanted to “detox” from smoking, referencing past experiences with recovery from heroin use to illustrate the relative difficulty of quitting smoking. “Deb” was a female participant in her early 40s and a single parent, as her ex-partner was deported out of the country several years ago. She lived with her two young daughters and had an older son who did not live with her.

Interviewer: *What type of help or support would you want for quitting?*

Deb: *I want to go into a hospital or a place of healing, in therapy or whatever, for five days. Get away from everything in my life and just leave me alone and just give me a week to detox from it, right? I’ll go to meetings of support or whatever, because I’ve been through addiction habits of other substances, right, and I’ve been able to kick those things…*

*I was using heroin for a short time in my life. And I was an IV drug user. This was before I had my son. And I quit that and I had gotten on methadone and I even quit that with no help and never picked up dope again. But yet, quitting cigarettes seems so impossible… there was a time where I went a few days without smoking, but I was around people who were not smoking and I had support and medical care. But I feel like I cannot do it unless I get a break from everybody, be by myself and build up the strength. When you’re under a constant stressful situation, it seems impossible.*

Life stress played a prominent role in participants’ perceptions regarding the difficulty of quitting. For example, Jasmine had dropped out of high school when she became pregnant and was now co-parenting three teenage sons with her ex-partner. She lived in Section 8 housing with her three sons. To make ends meet, she cleaned houses and did other jobs under the table in addition to her full-time job as an administrative assistant. She wanted to learn how to better cope with stress in order to break the stress-smoking cycle.

Interviewer: *What are your thoughts about quitting right now?*

Jasmine: *So I want to. I actually just met with a woman a few days ago to get a Y membership [referring to the YMCA, a nonprofit organization with locations across the U.S. that has workout facilities and offers fitness classes] for me and the family. I just need—I need a lot. I don’t know how the typical person stops smoking, but I feel like my life is always surrounded by stress. And the only way to really stop smoking is to remove that, and that’s like impossible. So I guess one would be me learning how to cope with the stress first. This way, when stress arises, then I’m able to cope with it enough where I don’t need a cigarette.*

Perhaps through prior quit attempts or other therapy, Jasmine had learned to identify stress as a trigger for smoking and the need for an alternate coping strategy for stress.

In this theme, the specific narratives along with the participants’ contexts reveal how the stress caused by socioeconomic disadvantage and housing instability were barriers to quitting and staying quit. When envisioning what would allow them to quit smoking, participants drew on a model of residential treatment programs for substance use disorder, which allow people to drastically change their current circumstances, even if only for a few days, to quit successfully. However, participants also acknowledged that such a change in one’s environment would require financial resources they may not be able to mobilize.

**Theme 5: Childhood adversity at intersection of current food insecurity and tobacco use**

Participants experienced important structural factors underlying shared vulnerability to food insecurity and smoking, as described in the overarching context of these results. Particularly, when participants described their initiation of and history with tobacco use (see Table 1 for relevant sample questions), adverse childhood experiences and trauma were commonly noted. These included childhood poverty and homelessness, involvement in the foster care system, and mental illness and substance use in primary caregivers. For example, “Jenny”, who lived in subsidized housing for older individuals and people with disabilities, explained that her addiction to cigarettes was a result of her childhood exposures:

*I grew up with parents that were smokers, alcoholics, so it was always around us. That’s why addiction and smoking started early with me. It was always around us. My father was very abusive so my mother ended up leaving my father… And it wasn’t until later that I realized you don’t give teenagers or your children beer and liquor and cigarettes and stuff. So I felt like my addictions were never my choice. Somebody else made the choice for me to be an addict, because a child doesn’t choose to be an addict.*

(Jenny, female participant in late 40s)

In contrast to Anne’s and Jan’s reliance on a logic of individual responsibility (in Theme 2), Jenny foreswears this understanding of addiction and asserted that, because they stem from childhood trauma, she could not be responsible for her addictions because children lack the capacity to fully choose their health behaviors. 

Like Jenny, Anne (the personal care attendant) experienced adverse childhood circumstances that informed her own parenting struggles. Anne was involved in the foster care system during her youth and experienced severe unmet basic needs while growing up. Her narrative clearly illustrates the challenges of raising a family in poverty.

*My mom, she had three daughters and she was trying to support us but we had to go without a lot of times. Like any new clothes or shoes that I got weren’t new. I had gotten them from friends at school. It’s just like, disheartening to feel that you just don’t have enough to get your basic needs. And so like when I wasn’t working so much because it was a good few months or more, and it was just really disheartening because it’s like my son would want something and I had to say no because I don’t have the money. I just don’t have the money for it. I’m now getting my hours back. But catching up is really hard. Trying to have a good birthday for my son, if it wasn’t for a good friend of mine, like my son probably wouldn’t have had a birthday. Yeah. It’s tough.*

(Anne, female participant in late 20s)

Anne alluded to the cyclical nature of her current food and economic insecurity, in which there was a sensitive ebb and flow in periods of sufficiency and scarcity due to a constantly changing schedule working at a fast food restaurant. Anne related her struggle to provide for her son to her own childhood experiences in which her mother was not able to provide for her basic needs. In this theme, co-occurring vulnerability to food insecurity and to smoking occurred across the life course and shaped participants’ current experiences of smoking addiction. This theme highlights the need to understand current struggles with food insecurity and tobacco use from a life course perspective that is cognizant of childhood exposures to adversity and trauma. Cessation efforts that fail to recognize early adversity and relevant life histories may be unable to address the deep roots of current challenges.

**Figure 1:**
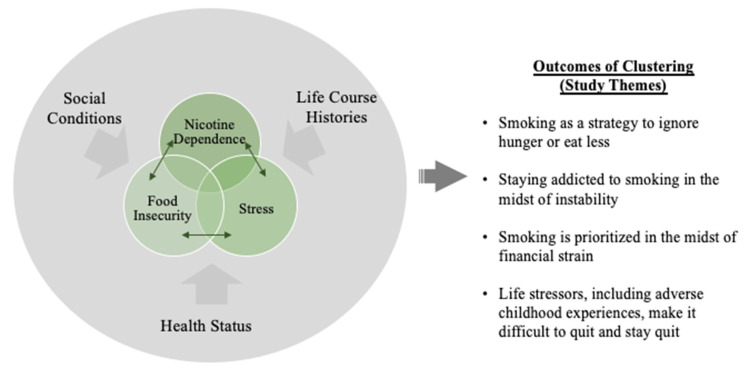
A Proposed Conceptualization of Clustering of Conditions Contributing to Nicotine Dependence, Food Insecurity, and Stress.

Our findings led to an initial conceptualization (Figure 1) that illustrates how social conditions, life course histories, and health status converge in a manner that generates shared vulnerability for food insecurity, nicotine dependence, and ongoing stress.

## 4. Discussion

This qualitative investigation explored the perceptions, lived experiences, and context of tobacco use and cessation in a sample of low-income adults who experience food insecurity in a largely rural setting in the U.S. In these narratives, the function and consequences of smoking were multilayered. Addiction to smoking occurred in the midst of life stress and ongoing instability, concerns about food sufficiency for themselves and their family, management of diet-sensitive chronic conditions, and strains caused by living with unmet basic needs such as housing. These findings highlight that cigarette smoking behaviors are complexly interwoven with experiences of socioeconomic and structural disadvantage, with important implications for addressing cigarette use disparities among people with low income. 

Researchers have argued that people engage in behaviors that are harmful to their health not because they do not know about the associated health risks but because of the varied and accumulated life constraints that make it difficult to enact behavior change [20]. Adding to the growing literature on the associations between food insecurity and tobacco use, which has been largely quantitative [11], this notion was reflected across the interviews and life course histories, as participants had a clear understanding of smoking’s negative impacts on their health, relationships, and finances but continued to smoke under conditions of considerable socioeconomic stress. 

These life stories emphasize that smoking and nicotine dependence must be addressed alongside food insecurity and stress since they are dynamically interacting conditions. As shown in Figure 1, social conditions, life course histories, and health status converge into a cluster, and these factors exacerbate one another in a cyclical fashion, with prior quantitative work suggesting that smoking exacerbates food insecurity, which exacerbates mental distress, which exacerbates smoking [21]. 

The themes described here can be understood as shared experiences among individuals living at the intersection of social conditions and life histories that produce stress, nicotine dependence via smoking, and food insecurity. Participants in this study relied on smoking as a constant source of stress relief in the midst of severe socioeconomic disadvantage and instability. Food insecurity was another stressful trigger for smoking, which reduced hunger. Adverse childhood experiences increased one’s vulnerability to addiction not just to cigarettes but other substances and made addictive behaviors more likely for some. Considered at a population level, the intersection of these social conditions drives tobacco use disparities and makes existing tobacco cessation programs less effective for those experiencing the highest need. Since increasing access to cessation resources has not reduced socioeconomic disparities in tobacco use, a health equity lens demands a more holistic approach to cessation that directs resources to the most marginalized individuals and addresses these intersecting social conditions. For instance, longer-term investments in policies and programs that can lift people out of poverty are needed to address a critical root cause of health disparities [22]. Furthermore, the field of tobacco cessation is beginning to recognize the value of screening for and attending to complex social needs as an added component of evidence-based cessation interventions [12]. Similarly, trauma-informed approaches are becoming more common elements of cessation programs [23]. 

The study findings should be interpreted in the context of some limitations. As participants were recruited from a U.S. state with higher tobacco taxes than the national average, it is possible that the financial burden of tobacco use is more pronounced than in states with relatively lower taxes. The sample was predominantly women, so examining differences in experiences by gender was beyond the scope of this initial investigation. Additional qualitative research on intersecting experiences of food insecurity and smoking is likely to further elucidate roles of geographic location, gender, and other related factors. It should be noted that the study interviews were conducted several months before the onset of the COVID-19 pandemic, which had broad impacts on food insecurity [24,25] and mental health [26,27] as well as tobacco use [28,29] across the population. While this research does not document the effects of COVID-19 in this community, ample evidence elsewhere suggests that the relationships and dynamics discussed here have only been exacerbated by the pandemic. 

## 5. Conclusions

This qualitative investigation elucidated complex and varied experiences of tobacco smoking among adults with food insecurity. Collectively, the themes speak to the need for tailored approaches to evidence-based tobacco cessation services and interventions that are designed to acknowledge and address the context in which smoking is perpetuated for people with unmet basic needs. Increasing access to evidence-based cessation treatments has been insufficient in and of itself and underscores the need to address food insecurity and related structural challenges faced by many low-income people who smoke. Health equity demands a focused effort to meet the specific needs of those for whom traditional approaches have been unsuccessful. For instance, this may involve direct food assistance and medical tailoring of meals for people as they quit smoking, particularly for those who also have diet-sensitive chronic conditions, coupled with greater access to nicotine-replacement therapies to manage acute symptoms of withdrawal. Efforts to help people who smoke identify alternate forms of stress management, for example, should directly address the role of socioeconomic stressors as triggers. Future research is needed to examine whether addressing food insecurity and related complex needs will yield improved cessation outcomes. More broadly, attention to the structural factors and early life adversity that underlie health behaviors and outcomes are needed to achieve health equity.

## Figures and Tables

**Table 1:** Sample Interview Questions and Probes.

Can you walk me through a typical day in your life? What is your current living situation? Who do you live with? What are current stressors in your life? What things do you most worry about? What is your general pattern of smoking in a typical day? What was yesterday like? Can you walk me through your history with tobacco, like at what age you first tried smoking and at what age you started to smoke regularly? Did family and others around you smoke while growing up? What do you like about smoking? What do you not like about smoking? How do cigarettes generally factor into your budget? How do you pay for your cigarettes? Are there any changes to how much or how often you smoke based on how much money you have? What are your thoughts about quitting? Have you ever tried to quit before? Can you tell me more about the circumstances around that? What supports would you want for quitting? You mentioned previously that you have worried about running out of food or that the food you bought didn’t last. Can you tell me more about your current food situation? What do you do to make ends meet?

**Table 2:** Characteristics of Study Participants.

Characteristics	*n* (%)
Gender	
Female	18 (78%)
Male	5 (22%)
Age range	
21–34	7 (30%)
35–49	8 (35%)
50–64	7 (30%)
65 or older	1 (4%)
Race and ethnicity	
African American / Black	3 (13%)
Hispanic or Latino	3 (13%)
White, non-Hispanic	15 (65%)
Another race or multiple races	2 (9%)
Education level	
Less than 12 years	3 (13%)
High school or GED	7 (30%)
Some college	3 (35%)
College degree or more	5 (22%)
Smoking characteristics	
Smokes daily	21 (91%)
Number of cigarettes in a typical day, M (SD, range)	15 (8, 1–35)
Food insecurity indicators	
Worried about running out of food	23 (100%)
Food didn’t last	20 (87%)

## Data Availability

Interview transcripts available upon reasonable request by contacting the corresponding author.
